# Occipital nerve block for headaches: a narrative review

**DOI:** 10.22514/jofph.2024.010

**Published:** 2024-06-12

**Authors:** William H. Arata, Rajiv K. Midha, Giustino Varrassi, Kelly Sala, Michael J. Plessala, Jared Brodtmann, Kylie Dufrene, Zachary Palowsky, Patricia Griffin, Shahab Ahmadzadeh, Sahar Shekoohi, Alan D. Kaye

**Affiliations:** School of Medicine, St. George’s University, St. George, Grenada; Paolo Procacci Foundation, 00193 Roma, Italy; School of Medicine, Louisiana Health Sciences Center at New Orleans, New Orleans, LA 70112, USA; School of Medicine, Louisiana State University Health Sciences Center Shreveport, Shreveport, LA 71103, USA; Department of Anesthesiology, Louisiana State University Health Sciences Center Shreveport, Shreveport, LA 71103, USA; Departments of Anesthesiology and Pharmacology, Toxicology, and Neurosciences, Louisiana State University Health Sciences Center Shreveport, Shreveport, LA 71103, USA

**Keywords:** Occipital nerve block, Greater occipital nerve block, Local anesthetic, Chronic headache, Migraine, Occipital neuralgia

## Abstract

The occipital nerve block involves the injection of a local anesthetic and possibly a corticosteroid near the occipital nerves at the base of the skull and aims at providing relief from chronic headaches by temporarily numbing or reducing inflammation around the occipital nerves. It has proven to be efficacious in treating chronic headaches, especially those that are refractory to medication; it is both diagnostic and therapeutic with symptom abatement from weeks to months. Occipital nerve blocks can be utilized alone or with standard-of-care therapy for various other headache conditions, such as cluster headaches, episodic headaches or chronic migraines. This review aims to provide an analysis of the world literature on the role of occipital nerve block (ONBs) in the treatment of headaches, including the procedure, efficacy and safety of ONB, and to elucidate the current understanding of ONBs. ONBs demonstrate diagnostic and therapeutic benefits with symptom abatement for weeks to months. Outcomes are generally well tolerated, with minimal side effects associated mainly with the injection process, including numbness, tingling and local discomfort. Greater occipital nerve blocks are an efficacious procedure to treat chronic headache symptoms, and future research should include additional longitudinal follow-up results and interactions of ONBs with other headache treatments.

## 1. Introduction

More than 15% of the population reports suffering from severe, debilitating headaches, making it the tenth most common health problem and the leading nervous system disorder. Between 1.4% and 2.2% of the global population contends with headaches for at least 15 days each month [1]. This painful condition significantly diminishes the quality of life and carries a substantial socioeconomic burden [2]. Consequently, managing headache pain is gaining traction as an increasingly prominent topic among specialists. Occipital nerve blocks are a growing interest in a treatment strategy for the management of chronic headache pain. The greater occipital nerve (GON) perforates the fascia beneath the superior nuchal ridge and lies medial to the occipital artery. The GON is commonly associated with occipital neuralgia, cervicogenic headaches, cluster headaches and migraine headaches [3]. A GON block can offer substantial pain relief as a primary treatment for headaches while other options are explored. The extent of symptom improvement often varies among patients and can be challenging to predict accurately [4]. This narrative review, therefore, describes GON blocks and their effectiveness for headache relief, safety and potential side effects.

## 2. Methods

This was a narrative review. The sources for this review are as follows: searching on PubMed, Google Scholar, Medline and ScienceDirect; using keywords: Occipital nerve block, greater occipital nerve block, local anesthetic, chronic headache, migraine, occipital neuralgia. Sources were accessed between December 2023 and March 2024.

### 2.1 Anatomy and greater occipital nerve block procedure

The GON is a sensory nerve that arises from the medial branch of the dorsal ramus of the second cervical nerve (C2) and may also receive fibers from the dorsal ramus of C3 [5, 6, 7]. The GON potentially pierces multiple muscles along its course. In 7.5% of cases, the GON pierces the inferior oblique muscle; in 45% of cases, it penetrates the trapezius muscle; in 90%, it penetrates the semispinalis muscle. When not penetrating the semispinalis, the GON runs medial to this muscle [5, 8, 9]. Compression, entrapment and irritation of the GON may occur at the locations at which it pierces these muscles along its course [5, 6, 7]. After penetrating the semispinalis muscle, the nerve meets the trapezius muscle and penetrates its fascia below the superior nuchal line of the occipital bone to meet the occipital artery, which it travels with medially [10, 11]. In most individuals, the courses of the two GONs are symmetric bilaterally; however, over 40% of individuals have asymmetry in the courses of the left and right GONs, meaning methods to identify the GON on one side do not necessarily correctly identify the position of the contralateral GON [12]. The GON then provides cutaneous innervation to the posterior scalp up to the vertex of the skull [5, 7]. It also provides sensation to the skin of the external ear and the skin superficial to the parotid gland [5]. The GON can elicit headaches in multiple ways, including compression in certain neck positions, as a result of neuralgia as a postoperative complication after cranial and posterior cervical surgeries, and, most commonly, idiopathically [5, 6, 7].

Blocking the GON with a local anesthetic can be a therapeutic approach to alleviate a variety of headaches in the occipital area, a procedure known as greater occipital nerve block (GONB).

The local anesthetics commonly used for GONBs are 2% lidocaine and 0.5% bupivacaine [13]. Additionally, for the treatment of cluster and cervicogenic headaches, anti-inflammatory steroids such as 40 mg/mL methylprednisolone or 2 mg/mL dexamethasone might be added for treatment of cluster headache [13]. However, the injection of local anesthetics plus corticosteroids has not been shown to further decrease migraine frequency or pain intensity when compared to the injection of local anesthetics alone [14, 15, 16]. The total injection volume per nerve block should not exceed 4 mL [13]. Lidocaine and bupivacaine can be combined [13]. No difference in patient response to GONB based on choice of local anesthetic has been demonstrated. Although bupivacaine is the longer acting anesthetic, injection of lidocaine, bupivacaine or a lidocaine/bupivacaine combination was not observed to affect patient response to treatment, duration of pain relief, or decrease in pain intensity [17, 18].

GONBs can be performed distally or proximally. To perform both a distal and proximal GONB, the patient can be seated or lying prone and must slightly flex the neck. For a distal GONB, to locate the GON, the occipital artery can be palpated about 2 cm lateral to the occipital protuberance, with the GON lying just medial to the occipital artery [12, 13, 19]. Alternatively, the GON can be located by palpating the occipital protuberance and the ipsilateral mastoid process. One-third (1/3) of the distance between these two points, from the occipital protuberance, superior to the superior nuchal line, lies the GON [20]. In addition to locating the GON *via* palpation, high-frequency ultrasonography with a frequency of 8–13 MHz can be utilized in both proximal and distal GONBs to locate the GONs and other superficial structures to more reliably prevent injury to or injection into the occipital artery during distal GONB. Ultrasound-guided location of the GON also allows for bilateral location of the two GONs, accounting for asymmetry in the courses of the two GONs. Utilization of ultrasonography to locate the GON has been shown to decrease complications and adverse effects as well as improve relief onset time and blockage success rates by exposing the location of possible nerve entrapment. In a distal GONB, scanning for the GON should begin midline 3–4 cm below the occipital protuberance and proceed laterally. Color doppler can be used to locate the occipital artery [21, 22, 23, 24]. After locating the GON, the area should next be cleaned with an alcohol swab, povidone-iodine or chlorhexidine [20]. Using a 23–25-gauge needle, insertion can be at an inferolateral approach or perpendicular to the skin until resistance is met [12, 20]. Next, withdraw the syringe 1 mm and aspirate to ensure the needle was not inserted into the occipital artery to prevent systemic injection of the anesthetic [12, 20]. The anesthetic can be injected directly into the GON or injected in a fan-like distribution. The fan-like distribution consists of 1 mL of anesthetic injected into the nerve, 1 mL lateral to the nerve, and 1 mL medial to the nerve [12, 20]. Alternatively, if pain is elicited while palpating to locate the GON, an injection can be performed at the site of maximal tenderness [20].

A proximal GONB is performed at the level of the C2 vertebra. High-frequency ultrasonography should be used to locate the GON and guide the injection. To begin, the US probe should be placed over the external occipital protuberance in transverse view and transferred caudally to visualize the C2 spinous process and then moved laterally to view the obliquus capitis inferior muscle. The GON can be located superficial to the obliquus capitis inferior, traversing the muscle from lateral to medial. The injection site should be cleaned with an alcohol swab, povidone-iodine, or chlorhexidine [20]. Injection should be performed using a 21–22 gauge needle inserted in-plane and progressed medially until near the GON in the fascial plane between the obliquus capitis inferior and the semispinalis capitis where the full contents of the syringe are injected [25].

Performing a GONB can improve pain 20–30 minutes after injection, and improvements can last from several weeks to several months [13]. Proximal and distal GONBs yield no significant differences in onset of pain relief or of degree of immediate pain relief, but proximal GONBs have been shown to yield longer-lasting pain relief compared to distal GONBs at 1–2 months [26, 27].

### 2.2 Primary and secondary headache etiologies and occipital nerve block

The success of an occipital nerve block can depend on many factors including the specific type of headache [28]. There are three main types of primary headache: tension-type, migraine and cluster [29]. Tension-type headaches are the most common primary headache subtype and prevalent neurological disorder in the general population [30]. The typical TTH attack presents with a non-pulsating, band-like distribution bilaterally. The etiology of tension-type headaches (TTH) is multifactorial, involving a combination of physical, psychological and environmental factors [30]. Migraines are primary headache disorders distinguished by usually unilateral headache pain and occasionally accompanied by incapacitating symptoms such as nausea, sensitivity to light and/or sound [31]. The previously prevalent vascular theory of migraines, proposing that the dilation of blood vessels caused migraine headaches and vasoconstriction was responsible for the aura of migraines, is now deemed obsolete [32, 33]. The current state of knowledge suggests that a primary neuronal dysfunction leads to a sequence of changes intracranially and extracranially that account for migraines [34, 35]. Cluster headaches are characterized by intense unilateral pain in the head and face, along with concurrent autonomic cranial symptoms on the same side (such as flushing, rhinorrhea and lacrimation) [36]. It is suspected that the first branch of the trigeminal nerve becomes activated and starts an inflammatory cascade, leading to pain [37].

In certain cases, occipital nerve blocks may also offer relief for secondary headaches, especially those such as cervicogenic headache, post-traumatic headache or post-dural puncture headache [38]. Cervicogenic headache is a type of referred pain commonly attributed to dysfunction in the upper cervical joints [39]. A double blind randomized controlled trial (RCT) showed that Greater and Lower Occipital Nerve block is effective to treat this pain [40]. Post-traumatic headache is a frequent sequela of traumatic brain injury either occurring as acute (<3 months) or persistent (>3 months) [41]. In a retrospective study of 10 postconcussive patients with headaches who were treated with greater occipital nerve blocks, 80% had a “good” response and 20% had a “partial” response, further illustrating how an occipital nerve block could treat various secondary headache causes [42]. The use of GONB has been demonstrated to be a quick, safe and effective method of alleviating headaches in pregnant patients who seek alternatives to classic pain relief medications to avoid teratogenicity. Importantly, GONB has also been demonstrated to be an effective treatment for post-dural puncture headache, a common complaint of pregnant patients who received spinal anesthesia during delivery. The headache typically varies with position, worsening when upright and improving when lying flat, often accompanied by neck stiffness, sensitivity to light, nausea or subjective hearing issues. The symptoms are thought to be caused by traction on pain-sensitive structures from low cerebrospinal fluid pressure (intracranial hypotension) following a leak of cerebrospinal fluid at the puncture site [43, 44, 45, 46, 47, 48].

### 2.3 Effectiveness

Several recent studies have explored the efficacy of greater occipital nerve (GON) blockade in chronic headaches, particularly for prophylactic applications in chronic or episodic migraine. A 2023 randomized controlled trial (RCT) looked at reduced headache days among chronic migraine patients randomized to four-weekly bilateral GON blockade with either lidocaine or saline for 12 weeks, explicitly focusing on outcomes during weeks 9–12. The treatment group was found to have a significantly greater change in the mean number of headache days from baseline compared to the control group (−7.2 *vs.* −3.0, respectively (*p* = 0.018)), along with a significant difference in the percentage of cases achieving ≥50% reduction in headache days from baseline (40.9% in the treatment group *vs.* 9.1 in the control group (*p* = 0.024)) [44]. Another RCT demonstrated the superiority of GON block with lidocaine, both with or without methylprednisolone, and in combination with concomitant standard-of-care topiramate in chronic migraine patients versus topiramate monotherapy. Patients in the lidocaine arm had a significant decrease in monthly migraine days at month three compared to the topiramate monotherapy group (−10.1 *vs.* −7.3, respectively (*p *< 0.001)), and similar results were demonstrated in the lidocaine plus steroid arm (−9.6 *vs.* −7.3 (*p* = 0.003)) [45].

Efficacy has been shown for prophylactic use of occipital nerve blockade in episodic migraine disorders as well. A 2021 retrospective study looked at 17 episodic migraine patients on prophylactic medication who received greater and lesser occipital nerve blockade, finding a significant reduction in median headache attack frequency during the month following treatment, in comparison with the month prior, from 5 to 2 (*p* = 0.001). There was also a reduction in pain days (12 pre-treatment versus 4 post-treatment (*p *< 0.001)) and Visual Analogue Scale (VAS) pain severity score (9 pre-treatment versus five post-treatment (*p* = 0.001)) [45]. One RCT showed statistically significant reductions in attack frequency at week 4 in episodic migraine patients who were randomized to receive GON blockade with lidocaine (−5.81 attacks per month, 95% CI (confidence interval) (−2.52–9.09)) or lidocaine plus triamcinolone (−5.69 attacks per month, 95% CI (−1.11–10.27)). In contrast, the reduction was not statistically significant in the cohorts receiving steroid alone or saline. Interestingly, no significant difference was seen between steroids or saline in attack frequency (*p* = 0.306). Furthermore, there was no significant difference between groups concerning headache severity and duration, in which all 4 groups demonstrated a significant change from baseline (*p *< 0.001 and *p* = 0.001, respectively). The authors suggest that a certain degree of improvement in these patients may be attributable to GON compression after the injection, thus explaining the universally significant reduction in severity and duration seen in all four groups, including saline [49]. There is also a growing body of evidence supporting the use of GON blockade in cluster headaches, particularly in refractory populations. A CT (computed tomography)-guided technique for GON blockade with lidocaine was studied in patients with cluster headaches refractory to standard oral therapy. Clinical success was characterized as a >50% reduction in headache severity on the VAS and was evaluated at 1, 3 and 6 months. Clinical success was achieved in 81% of cases, with 69% still demonstrating a response at three months and 31% at six months [50]. A 2017 prospective study enrolled patients with both episodic and chronic cluster headaches not adequately treated with standard abortive therapy or controlled by prophylactic treatment. Blockade of the ipsilateral greater occipital nerve was performed using bupivacaine plus triamcinolone, and patients were followed for complete or partial resolution of migraines. 84 of 101 enrolled patients (83.2%) experienced a response to treatment at day 1, with a mean duration of response of 27.3 ± 26.2 days. Significant reductions in attack frequency were also seen at day 7 in both groups (*p *< 0.001) [51].

Typically, GON blockade is performed blindly, targeting the distal GON using the superior nuchal line as a landmark for injection. However, more recently, an ultrasound (US)-guided approach has begun to be employed in clinical practice, visualizing the proximal GON near its origin at the C2 level for a more selective approach. There are a limited number of RCTs demonstrating similar or improved outcomes, namely durability of response, using the US- guided technique versus landmark-guided in cervicogenic headache and chronic migraine [26, 52]. Additionally, a recent single-center retrospective study found no extra benefit when the clinic added distal level GON block to their standard clinical protocol employing US-guided proximal GON block in chronic migraine patients, demonstrating no reduction in benefit by blocking the proximal GON instead of distal [53]. Another retrospective study compared reduction rates of severe attacks, total analgesic use and triptan use between migraine patients receiving GON blockade with either a blind or US-guided technique, finding similar results between the two groups [54]. The vast majority of studies on GON blockade in chronic headache have been conducted using the classic blind approach, but efficacy data supports the use of this more modern US-guided technique, and it should be considered for chronic migraine patients and certainly warrants further study.

### 2.4 Safety

GON blockade is generally a safe and well-tolerated treatment, with most adverse effects being mild and transient. The most common adverse effects are related to the steroid component if used in the block procedure or the injection itself. In a study that randomized chronic migraine patients to receive injections of either lidocaine or saline, a nearly identical safety profile was noted between the two arms. 72.7% of patients in the lidocaine group reported one or more adverse events (AEs), compared to 68.2% in the saline group and 63.6% of patients in both groups, demonstrating a negligible difference in the safety profile of the two groups. The three most common AEs in both arms were injection site bleeding, pain and swelling and resolved within a few days [44]. One recent trial also reported transient (<48 h) head numbness in 8% of patients receiving a GON block [55]. In another study, patients received GON blockade with either lidocaine or lidocaine plus methylprednisolone. These patients reported dizziness, and vasovagal syncope as adverse events in addition to injection site complaints; however, both occurred more frequently in the patients that included steroids [45]. This is consistent with other trials that administer GON blockade with local anesthetic, and steroids report a greater number and variety of AEs. One case of Cushing Syndrome was written in 2001, where a woman showed stigmata of Cushing Syndrome after receiving six bilateral GON blocks with 40 mg triamcinolone over three months [55]. To our knowledge, this is the first and only report of Cushing syndrome caused by occipital nerve blockade, and precautions should always be taken when administering corticosteroids longitudinally. One RCT reported having to halt patient recruitment in groups receiving triamcinolone due to intolerable AEs, such as cutaneous atrophy and localized alopecia at the injection site, noting in their discussion that such effects had not previously been reported in RCTs but were consistent with known adverse effects of local corticosteroid injection [49]. Taking into consideration that their data did not demonstrate a difference in efficacy with or without triamcinolone, albeit with a relatively small sample size, the risks and benefits of steroid use in the GON block procedure may warrant further study. Additionally, there is limited evidence comparing the safety profiles of blind and US-guided procedure techniques, but there are RCTs demonstrating similar AE data between the two [26, 52]. Nevertheless, data from these recent studies remains consistent with the evidence suggesting that GON blockade is typically well-tolerated and most adverse effects of the procedure prove to be transient.

## 3. Discussion

Recent U.S. data shows that chronic headache-related costs due to loss of productivity represent around 70% of total CM (chronic migraine)-related costs [56]. In most cases, these chronic headache symptoms present idiopathically, which has historically limited our ability to provide targeted and effective relief for those afflicted by this disorder [3]. GON blocks represent a therapeutic intervention designed to address chronic headaches. The observable outcome of a GON block is evident within the trigeminovascular system, a critical physiological pathway intricately involved in the manifestation of migraines and cluster headaches [57]. This procedure involves the percutaneous administration of a local anesthetic, often augmented with a corticosteroid near the GON, aiming to interrupt nociceptive transmission. The administration of a local anesthetic injection to the GON, a branch of the upper cervical nerve, hinders afferent stimuli from regions innervated by the GON. This impedes the sensitization of C2 dorsal horn convergent neurons by diminishing their input [58]. This intervention is most useful in patients for whom traditional pharmacological therapy is ineffective or deemed unsafe because of pre-existing conditions. The procedure itself was heavily scrutinized in a systematic review and meta-analysis that looked to prove the efficacy of the nerve block for patients suffering from migraine headaches [59].

While there has been positive evidence for GONBs showing effectiveness in reducing pain related to cluster headaches, there has not yet been a statistically significant study analyzed to indicate that the procedure is as effective on primary headaches as it is on chronic or migraine headaches [59].

Our analysis of several recent studies yielded positive results suggesting that patients who underwent GONB saw significant decreases in chronic pain related to migraines without any major associated side effects. A meta-analysis and systematic review in 2019 also corroborates these results [60]. Mild adverse effects related to the procedure were found to be related to tenderness at the injection site, distension of the abdominal cavity, numbness and tingling near the injection site, and redistribution of fat [61]. Some dizziness and vasovagal syncope were reported in addition to injection site complaints, however, these symptoms were not frequently reported and only occurred in patients who received both the lidocaine and steroid combination injection [45].

A major limitation has been that many of the studies performed to analyze GON blocks have been reliant on relatively small sample sizes. This overall has limited the ability of neurologists and pain management specialists to mass-administer this procedure to patients with confidence. One narrative review found a surprising lack of standardization in the literature regarding the dose of anesthetic medication or steroid medication if there is a need for bilateral injections, procedural technique or frequency of doses [57]. The procedure is commonly used among clinicians, but its standardization seems to be a limiting factor in its true efficacy.

However, Table 1 produced here represents a systematic review that found a few answers to some of these shortcomings. A retrospective study that examined 17 patients aged 26–57 with chronic migraines who received occipital nerve blocks in 3 different sessions found that bilateral nerve blocks are not only safe for migraine prevention but also an effective therapy. Other studies in the table further suggest that GONBs are effective treatments for headache prophylaxis, migraines and tension-type headaches. Some questions were left unclear as, for example, no clinical significance was found between lidocaine with steroid treatment versus a lidocaine placebo. Uncertainty about which medications prove the most efficacious injecting, therefore, remains. Now that the GONB is proven to be efficacious at relieving chronic headache symptoms across a myriad of various headache phenotypes, deeper investigation into the optimal combination of injected medications is warranted.

**Table 1.** Clinical efficacy and safety of occipital nerve blocks.

Author (yr)	Groups Studied and Intervention	Results and Findings	Conclusions
Study 1: Hasan Hüseyin Kır [62] (2022)	122 cases with primary headache disorders (86 with migraine headache, 33 with tension-type headache (TTH), 1 with cluster headache, and 1 with trigeminal neuralgia) resistant to oral medication, received a bilateral 2% lidocaine injection to the greater occipital nerve. The blocking procedure was applied four times, once a week in the first month and once a month in the 2nd and 3rd months, for 6 sessions. Clinical data was evaluated pre- and post-procedure.	The frequency of headache attacks decreased from 13 to 5, and the pain scale score decreased from 9 to 5 after the third month within the migraine group. Likewise, headache attacks reduced from 17 to 7, and the pain scale score decreased from 8 to 4 in the TTH group. Significant improvements were seen in all parameters for both groups. Only one patient, a 32-year-old female patient with a migraine, developed hypotension and dizziness. No significant complications were reported in other cases.	The GON block emerges as a minimally invasive approach for the treatment of headache disorders in patients resistant to oral medication, specifically those with migraine and TTH.
Study 2: Malekian *et al*. [49] (2022)	Fifty-five patients suffering from chronic episodic migraines received either triamcinolone, lidocaine, triamcinolone plus lidocaine, or saline and followed for four weeks at 1-week intervals.	Groups 2 and 3 (lidocaine and triamcinolone with lidocaine) showed a significant decrease in the frequency of migraines from baseline (*p* = 0.002, *p* = 0.019, respectively). Three patients reported adverse events (cutaneous atrophy and alopecia) with a possible correlation with triamcinolone.	Patients who received lidocaine, either alone or combined with triamcinolone, had fewer headaches than the other groups or control. This study supports the use of lidocaine for greater occipital nerve blocks when considering treatment options.
Study 3: Chowdury *et al*. [44] (2023)	Forty-four patients suffering from chronic migraines were randomized to either an active group or placebo and received four-weekly bilateral greater occipital nerve blockade with either 2 mL of 2% (40 mg) lidocaine or 2 mL of 0.9% saline (control group) injections for 12 weeks.	Reduction in mean number of migraine days was greater for the active group (−6.4 days (95% CI: −9.8 to −5.8)) compared with placebo (−1.8 days 95% CI: (−5.1 to −1.6)) with the LSM difference of −4.7 days (95% CI: (−7.7 to −1.7); *p* = 0.003). 40.9% of cases in the active group showed ≥50% reduction in headache days compared to 9.1% of cases receiving a placebo (*p* = 0.024).	Four-weekly greater occipital nerve blockade with 2% lidocaine (12 weeks) was superior to placebo in reducing the average number of headaches and migraine days in cases with chronic migraine.
Study 4: Cvetkovic *et al*. [67] (2021)	10 participants with a history of two or more previous unsuccessful pharmacological preventive treatments for chronic migraines were enrolled in a 24-week, randomized, double-blind, placebo-controlled, crossover trial and underwent treatment with bilateral greater occipital nerve block with lidocaine plus betamethasone or lidocaine plus saline with a 4-week interval wash-out phase between the 8-week crossover periods.	No difference between lidocaine plus betamethasone and lidocaine plus saline on the reduction of monthly migraine days was observed (*p* = 0.147; 95% CI between 0.6 and 3.7 days).	There was significant difference between lidocaine plus betamethasone and lidocaine plus saline for either primary or secondary efficacy endpoints, including migraine days, headache days, acute medication days and headache intensity. Overall, the procedure was well-tolerated.
Study 5: Gunes and Ozeren [12] (2021)	17 participants aged 26–57 with history of chronic episodic migraines and currently using migraine prophylaxis received occipital nerve blocks (ONB), (on days 0, 15 and 30) in the first 4-weeks in this retrospective study. Patients were asked to keep a monthly headache diary.	The median headache frequency of the cases was 5/month (range, 4–14) before therapy, whereas it was 2 (range, 0–6) after ONB (*p* = 0.001). The median duration of pain before ONB was 12 (range, 6–14) days/month, and it was 4 (range, 0–9) days/month after ONB (*p *< 0.001). No side effects were reported.	ONB could be a treatment option in episodic migraine cases without aura who are resistant to oral medicines.
Study 6: Chowdhury *et al*. [45] (2022)	125 patients with consecutive adult chronic migraine were randomized to one of three treatment arms: receiving topiramate monotherapy (100 mg/day), initial injection of topiramate plus greater occipital nerve (GON) block with 40 mg lidocaine and 80 mg methylprednisolone followed by monthly lidocaine injections for two months, or topiramate plus GON block with 40 mg lidocaine monthly for three months. A number of monthly migraine days was assessed up to month three.	Patients receiving combination therapy with topiramate and GON block with lidocaine plus steroids or GON block with lidocaine alone achieved significant reductions in a number of migraine days at three months (−9.6 *vs.* −7.3 days; *p* = 0.003) and (−10.2 *vs.* −7.3 days; *p *< 0.001), respectively. Also, patients showed ≥50% reduction in monthly migraine days in the lidocaine + steroid arm (71.4% *vs.* 39%; OR = 3.9; 95% CI (1.6–9.8); *p* = 0.004) and lidocaine arm (62.4% *vs.* 39%; OR = 2.7; 95% CI (1.1–6.7); *p* = 0.034) *vs.* topiramate monotherapy. No serious adverse were reported.	GON blockade with lidocaine +/− steroid is safe and effective when performed in combination with first-line topiramate therapy for prophylaxis of migraine headaches in chronic migraine patients, with a significantly greater reduction in monthly migraine days and a significant increase in the proportion of patients achieving a ≥50% reduction in monthly migraine days.

*CI: Confidence interval; LSM: Least squares mean; OR: Odds ratio.*

Most study designs analyzing the effectiveness of GONBs are determined to be retrospective cohort studies. There is consistency in the data; however, when a meta-analysis that examined average pain scores after a GON block was performed, patients across the studies saw reductions in their chronic pain levels after the completion of a GONB [62]. While consistency is a benefit to the stability of the findings across studies, it would be a crucial point of further research to investigate if the results remain consistent across different types of studies, such as prospective cohort studies rather than retrospective cohort studies.

In practicality, the GON block is very well suited for clinical use. It does not require general anesthetics, pre-operative instructions for the patient, significant time to perform, or notable adverse effects that would inhibit patients from returning to their lives. For patients who struggle with consistent pharmacological intervention or unwanted side effects related to medications, this therapy is found to be an effective alternative. Also, this procedure can be sought out by patients who wish to receive immediate relief for chronic pain related to the rapid onset of subcutaneous medication. Chronic headache symptoms, as previously stated, most commonly present idiopathically with no known cause. This makes the disorder notoriously challenging to treat, as specific therapies are less commonly available to patients.

The GON block and its proven efficacy could prove revolutionary for many patients with underlying, mal-understood pathophysiologic conditions that have not been effectively targeted by previous therapies. One such condition is post-dural puncture headache. The most effective current treatment is an epidural blood patch (EBP) [63]. However, this is an invasive method with potential morbidity [63]. While EBP is the gold standard, GON has been shown in various cases and one retrospective study to be a less invasive, easier and more effective method to treat post-dural puncture headache and could be considered before applying the EBP [64]. More studies are needed comparing the outcomes of the GON block before receiving the EBP to identify a new gold standard. Further research should also be directed towards optimizing or improving the nerve block procedure itself as there is some discrepancy in the technique. Most studies and summary articles use palpable physical landmarks to assess the location of injection, however, the recommended location for injection varied [65, 66]. Clinicians will want to understand the most effective technique, which medications patients respond to at higher rates, and how often the procedure should be performed for their chronic patients. Standardization of these components will build on the research in place that states that this is an evidence-based approach worth administering to patients struggling with chronic migraine headaches. Considering how high the incidence of patients with chronic migraines is, along with other forms of chronic neuralgias, this therapy should be utilized clinically and further studied.

Patients who received a GONB showed a significant reduction in the mean number of headache days per month compared to those who did not, with many of these patients achieving >50% reduction in headache days from their clinical baseline [44, 67, 68, 69]. Patients receiving GONB, with or without supplemental steroids, were proven to have achieved a significant reduction in monthly migraine days three months post-injection as compared to the control group [44]. Specifically catering to patients experiencing migraine symptoms, GONBs were proven to significantly reduce patients’ median migraine frequency during the month following injection [12]. Additionally, these studies confirmed GONB efficacy in controlling refractory cluster headache symptoms compared to their controls. In all studies reviewed, a consistent result emerged, namely that the GONB consistently demonstrated effectiveness in alleviating symptoms of chronic headaches (Table 1).

## 4. Limitations of our review

This review focused predominantly on retrospective cohort studies, which opens the possibility that conclusions are subject to hindsight biases. Prospective cohort studies regarding the use of GONB for treating chronic headaches are a potential area for further research, and exploration is needed to fortify the results outlined in this review. Furthermore, most of the studies in this review followed patients for less than one year after their respective GONB procedures, which limits the ability of this review to speak of GONB as a potential long-term treatment or curative agent for chronic headache symptoms.

## 5. Conclusions

Chronic headaches are an increasingly common, debilitating and notoriously hard-to-treat disorder. It is, therefore, prudent to explore the GONB as a productive, non-invasive and overall safe treatment option for patients whose pain is not controlled by conventional oral medications. While this review has concluded that the GONB is an efficacious procedure to treat various chronic headache symptoms, future research can and should be conducted to deduce the most optimal cocktail for these injections to maximize patient results. Research can and should also be expanded to follow patients for more than one year after their initial GONB procedure, and, to ultimately, establish an interval recommendation for patients regarding how often these blocks should be administered. As with all therapies, interactions with other headache treatments, such as oral medications, should be further explored for maximal treatment efficacy and safety if used concurrently.
